# The Avon Longitudinal Study of Parents and Children - A resource for COVID-19 research: Questionnaire data capture April-May 2020

**DOI:** 10.12688/wellcomeopenres.16020.2

**Published:** 2020-11-20

**Authors:** Kate Northstone, Simown Howarth, Daniel Smith, Claire Bowring, Nicholas Wells, Nicholas John Timpson

**Affiliations:** 1 Population Health Science, Bristol Medical SChool, University of Bristol, Bristol, BS8 2BN, UK; 2 Bristol Dental School, University of Bristol, Bristol, BS1 2LY, UK; 3 MRC Integrative Epidemiology Unit, Department of Population Health Sciences, Bristol Medical School, University of Bristol, Bristol, Bristol, BS8 2BN, UK

**Keywords:** ALSPAC, Children of the 90s, birth cohort study, COVID-19, coronavirus, online questionnaire, mental health

## Abstract

The Avon Longitudinal Study of Parents and Children (ALSPAC) is a prospective population-based cohort study which recruited pregnant women in 1990-1992. The resource provides an informative and efficient setting for collecting data on the current coronavirus 2019 (COVID-19) pandemic. In early March 2020, a questionnaire was developed in collaboration with other longitudinal population studies to ensure cross-cohort comparability. It targeted retrospective and current COVID-19 infection information (exposure assessment, symptom tracking and reported clinical outcomes) and the impact of both disease and mitigating measures implemented to manage the COVID-19 crisis more broadly. Data were collected on symptoms of COVID-19 and seasonal flu, travel prior to the pandemic, mental health and social, behavioural and lifestyle factors.

The online questionnaire was deployed across parent (G0) and offspring (G1) generations between 9 ^th^ April and 15 ^th^ May 2020. 6807 participants completed the questionnaire (2706 original mothers, 1014 original fathers/partners, 2973 offspring (mean age ~28 years) and 114 offspring partners). Eight (0.01%) participants (4 G0 and 4 G1) reported a positive test for COVID-19, 77 (1.13%; 28 G0 and 49 G1) reported that they had been told by a doctor they likely had COVID-19 and 865 (12.7%; 426 G0 and 439 G1) suspected that they have had COVID-19.  Using algorithmically defined cases, we estimate that the predicted proportion of COVID-19 cases ranged from 1.03% - 4.19% depending on timing during the period of reporting (October 2019-March 2020).

Data from this first questionnaire will be complemented with at least two more follow-up questionnaires, linkage to health records and results of biological testing as they become available. Data has been released as: 1) a standard dataset containing *all* participant responses with key sociodemographic factors and 2) as a composite release coordinating data from the existing resource, thus enabling bespoke research across all areas supported by the study.

## Introduction

The coronavirus disease 2019 (COVID-19) pandemic is a rapidly developing global health challenge. There is marked heterogeneity in disease prevalence, severity and outcome both within and across populations. In part, this may be driven by the interplay between environmental, social and host factors such as age and pre-existing comorbidities which predispose or protect against infection or modify disease outcomes. Understanding this interplay requires studies with detailed environmental, health, lifestyle, and biological data – ideally measured within the context of longitudinal data and with prospective collection opportunities.

Alongside the health implications of the virus itself, the response to the pandemic is likely to affect health and wellbeing. Mitigation measures have resulted in far-reaching changes to daily activity which are likely to have an impact on nearly all aspects of work, family life, recreation and, potentially, health. The nationwide ‘lockdown’ strategy implemented on 23 ^rd^ March 2020, for example, meant that UK residents were only allowed to leave the house to: 1) buy basic necessities, 2) exercise once a day, 3) attend to a medical need or care for a vulnerable person 4) travel to and from work that could not be done at home ^1^. These strictest measures were in place for several weeks, with easing beginning in early May, schools returning in June and many non-essential shops opening their doors again in mid-June. Any adverse effects of these mitigation strategies may themselves be heterogeneous, with certain groups at higher risk of adverse effects. Understanding the effect of mitigation strategies on health and identifying the social, environmental, or other factors which help reduce their impact will be an important part of planning both ongoing and future COVID-19 mitigation strategies as the pandemic develops.

The Avon Longitudinal Study of Parents and Children (ALSPAC) is a unique three-generational study, comprising ‘G0’: the cohort of original pregnant women, the biological father and other carers/partners; ‘G1’: the cohort of index children and ‘G2’: the cohort of offspring of the index children. The study has a wealth of biological, genetic and phenotypic data across these generations ^2– 5^. ALSPAC has an opportunity to capture information across key parts of the population in light of the COVID-19 pandemic – in particular the contrast between those in higher risk (G0 mean age: ~58years) and lower risk (G1 mean age: ~28yrs) groups. We were well placed to collect data quickly using our existing infrastructure for online data collection.

The wider COVID-19 data collection in ALSPAC will include data from three main sources: self-reported data from questionnaires, data from clinical services based on linkage to medical and other records and though biological samples. The data from these sources are intended to be complementary and help address different potential research questions around COVID-19. This data note describes the data collected via our first online questionnaire between 9 ^th^ April and 14 ^th^ May 2020 and provides a summary of the participants who responded. Updates on subsequent questionnaires and other sources of data will be released as these additional datasets are made available.

## Methods

### Setting

ALSPAC is an intergenerational longitudinal cohort that recruited pregnant women residing in Avon, UK with expected dates of delivery 1 ^st^ April 1991 to 31 ^st^ December 1992 ^2, 3^. The initial cohort consisted of 14,541 pregnancies resulting in 14,062 live births and 13,988 children who were alive at 1 year of age. From the age of seven onwards, the initial sample was bolstered with eligible cases who had originally failed to join the study and there were subsequently 14,701 children alive at 1 year of age following this further recruitment ^4^. Please note, the study website contains details of all the data that is available through a fully searchable data dictionary and variable search tool.

In response to the COVID-19 it was necessary to develop a data collection strategy which was practical, would yield data quickly and could be updated and repeated. For these reasons, we chose to use an online only data collection approach for this, restricting our invites to those participants with a valid email address (and coordinated with a systematic communications/outreach campaign). The questionnaire was developed and deployed using REDCap (Research Electronic Data CAPture tools ^6^); a secure web application for building and managing online data collection exercises, hosted at the University of Bristol.

### Content design

Content was initially developed internally, we then consulted with a network of 16 UK and international longitudinal population studies and partners through a process facilitated by Wellcome (see acknowledgements). This resulted in a core set and a recommended set of questions about health, behaviour, social, economic and environmental impact of COVID-19. Whilst we were able to align many features of the ALSPAC questionnaire and the Wellcome core questionnaire, there are some areas of divergence. This is because the ALSPAC questionnaire was deployed while the Wellcome core questionnaire was still being finalized, and because we chose to use mental health measures that we have used previously to facilitate longitudinal analyses rather than the Wellcome recommended measures. It is worth noting that ALSPAC is the contact point for using the Wellcome questionnaire and we can provide data dictionaries in either REDCap or Qualtrics (via Generation Scotland) on request. This work is therefore part of a coordinated effort to generate and promote a Wellcome Trust supported core questionnaire. This is now complete and available and access to this can be organised through ALSPAC. The questionnaire has not been formally validated, however, extensive testing by our ethics committee, participant advisory group and ALSPAC staff led to clarity of wording and ensuring REDCap functionality worked as expected.

The questionnaire included 4 sections, and captured information on the following:

A. General health, recent travel and seasonal symptoms
  • Conditions making people high risk
  • Frailty assessed using PRISMA -7 ^7^
  • Regular medications (prescription and over the counter)
  • Home country and travel outside that country since October 2019
  • Symptoms of COVID-19 and negative control symptoms since October 2019
  • Tested/diagnosed with COVID-19
B. Behaviour as a result of COVID-19
  • Self-isolation and reasons why
  • Behaviour changes prior to lockdown
  • Lifestyle changes since lockdown
  • Social contacts and methods of communication
C. Impact of the pandemic
  • Worries during the pandemic
  • Depression assessed using the Short Moods and Feelings questionnaire (SMFQ; 8)
  • Anxiety assessed using the General Anxiety Disortder-7 questionnaire (GAD7; 9)
  • Well-being assessed using the Warwick-Edinburgh Mental Wellbeing Scales (WEMWBS; 10)
D. About you during the pandemic
  • Understanding of official guidance on COVID-19
  • Time spent talking/reading about or listening to information about COVID-19
  • Living arrangements
  • Healthcare worker and keyworker status
  • Effect of pandemic on plans to have children (G1 only)
  • Free text inviting participants to provide details of other ways they have been affected by the pandemic

The final questionnaire (REDCap PDF) used is available with the associated data dictionary (which includes frequencies of all variables that are available) and both are available as extended data ^11^.

### Invitation and reminder strategy

Between the 9 ^th^ and 15 ^th^ April 2020, all participants (G0, G1 and G1 partners enrolled as part of G2 (children of the Children of the 90s ^5^) for whom we had an active email address were sent an invitation to complete the questionnaire. Participants were not contacted if our administrative database record indicated that they were deceased, had withdrawn from the study, had declined further contact or had declined questionnaires. The questionnaire survey was live on the online platform for just over one month. After 2 weeks, any non-responders were sent a reminder email to complete the questionnaire. In addition, traditional (print, radio, tv) & social media (Facebook, Instagram and Twitter) were used to inform participants that the questionnaire was live, asking them to contact us if they had not received it and to encourage completion. These communication channels were also used to encourage re-engagement of friends and family back into the study. Unlike our standard questionnaires (usually completed annually) we did not provide any incentive for completion; however, we did offer a prize draw (three prizes of £100) for those who completed their questionnaire by 11 ^th^ May.

### Response rate

**Figure 1.**
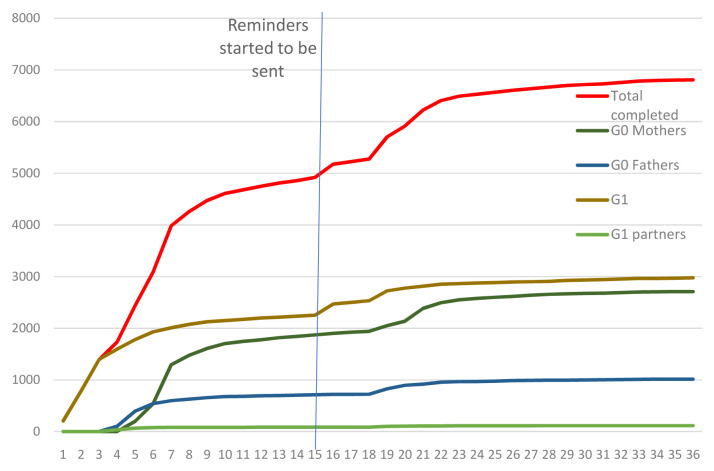
Completion rate by number of days questionnaire was live. Invitations were sent in batches over several days - G0 invitations did not start going out until 3 days after the questionnaire was live.

A total of 12,520 invitations were sent out and responses were received from 6811 participants (overall response rate of 54%). Over 4,000 participants completed the questionnaire within the first week of the invitation (see Figure 1), this represents a snapshot of participant experience around 3 weeks after the start of ‘lockdown’. The questionnaire was closed on 15 ^th^ May 2020.

Overall, female participants were more likely to respond ( Table 1), and this was particularly true of the younger generation. A potential explanation for the difference in male response rates between younger and older participants we note that G0 fathers included in this data collection exercise are already relatively engaged with the study as they had to enrol in their own right in 2011 as part of our ‘Focus on Fathers’ data collection (previously they were only invited to participate via the mother). The response from G1 males was disappointing and we aim to re-engage this group using targeted social media and other activities in the future. Table 1 summarises the response rate within each group organised by cohort structure.

Characteristics of responders according to key variables that will be released with the complete dataset can be seen in Table 2.

**Table 1.** Number of participants who were eligible and who responded to the first COVID-19 questionnaire.

Cohort Group	Eligible ^1^	Responded ^2^
G0 Mothers	4590	2706 (59%)
G0 Fathers/partners	1803	1014 (56%)
G1 Offspring daughters	3617	2126 (59%)
G1 Offspring sons	2225	847 (38%)
G1 Offspring partners (female)	103	63 (61%)
G1 Offspring partners (male)	182	51 (28%)
**TOTAL**	**12520**	**6807 (54%)**

^1^valid email address, marked as contactable for questionnaires ^2^Proportions of those invited (i.e. eligible)

**Table 2.** Summary of key characteristics for those who responded; n (%) for categorical variables, mean (sd) for BP and median (Inter-quartile range) for BMI.

	Mothers	Fathers/ partners	Offspring	Offspring partners
Age (years)	57.9 (4.44)	60.8 (5.17)	27.6 (0.54)	29.9 (4.37)
Latest BMI ^1^	25.4 (22.9, 28.7)	26.9 (24.9, 29.1)	23.4 (21.1, 26.9)	26.5 (23.3, 30.4)
Latest Systolic BP ^1^	119.4 (14.10)	132.9 (13.77)	115.2 (10.83)	116.3 (12.44)
Latest Diastolic BP ^1^	70.6 (9.33)	77.2 (9.03)	66.7 (7.77)	65.9 (9.97)
Education level ^2^ ≥A level	1385 (53.7%)	674 (70.4%)	1793 (77.8%)	26 (60.5%)
Ethnicity ^3^ White	2533 (98.4%)	948 (99.2%)	2559 (96.6%)	Not available

^1^Data taken from the most recent clinic that individual attended where available ^2^Data taken from pregnancy questionnaires for G0 and from most recent questionnaire for G1 where available ^3^Data taken from pregnancy questionnaires for all

### Key results

Participants were asked whether they thought they have had COVID-19. Options were: ‘Yes, confirmed by a positive test’, ‘Yes, suspected by a doctor but not tested’, ‘Yes, my own suspicions’ or ‘No’. Overall 8 respondents reported that they had tested positive to COVID-19 (when combined with participant group this information is potentially disclosive it will therefore be combined with ‘yes, suspected by a doctor’ to create a new category in the released datasets ‘Yes, tested positive or suspected by a doctor’). Table 3 summarises the responses to this question by cohort structure.

**Table 3.** Participant response to whether they have had COVID-19.

	G0 - parents	G1 – offspring (+partners)	Total
Yes, positive test	4 (0.01%)	4 (0.01%)	8 (0.01%)
Yes, doctor suspected, no test	28 (0.76%)	48 (1.56%)	76 (1.12%)
Yes, own suspicions	426 (11.5%)	439 (14.2%)	865 (12.8%)
No	3229 (87.4%)	2584 (83.8%)	5734 (84.6%)

Menni and Colleagues ^12^ recently published a model combining symptoms to predict ‘probable infection’ using data collected from an app-based symptom tracker ^13^. This algorithm uses four symptoms: loss of smell and taste, severe or significant persistent cough, severe fatigue and skipped meals (coded as 1 if present and 0 otherwise), together with age and sex (1 male; 0 female). We had slight difference in wording and thus the algorithm (using the same weightings) applied was as follows:

-1.32 - (0.01 x age) + (0.44 x sex) + (1.75 x loss of loss of smell *or* taste)
  + (0.31 x *new* persistent cough) + (0.49 x severe fatigue)
  + (0.39 x decreased appetite).

**Figure 2.**
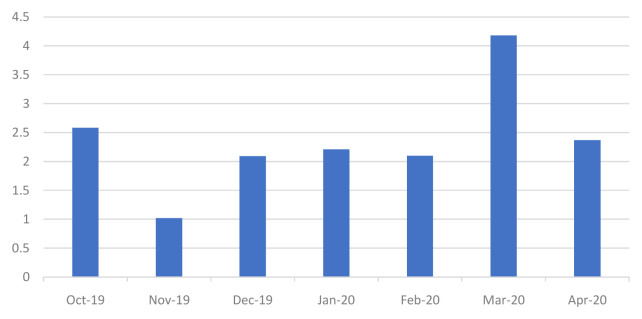
Predicted cases (% of population) of COVID-19 per month according to symptoms reported for those months using Menni et al algorithm ^12^.

Probable COVID-19 cases were obtained by applying an exp( *x*)/[1+(exp( *x*)] transformation and coding values >0.5 as probable cases. We applied this algorithm to our monthly symptom data and the number of predicted ‘cases’ each month are shown in Figure 2. We used Stata v.15.0 and our Stata do file is available as extended data ^11^. Peak predicted cases were seen in March (4.19%) with similar proportions seen in all other months (2.10% - 2.38%), except November which was substantially lower (1.03%). Our figures were consistently lower than the 5.36% of responders to the app ^12^ who were reported as likely being infected by the virus. It should be noted that Menni *et al*. developed their algorithm based on data collected in the spring of 2020 when COVID-19 levels were at their highest. We have applied the algorithm to data collected prior to this and demonstrate a predicted prevalence of between 1 and 2.5% in 2019. Whilst, COVID-19 may have been present in the UK population during this time, it is more likely that symptoms reported by participants during this period were as a result of other respiratory viruses and these predicted cases should therefore be interpreted with caution. Table 4 summarises the predicted cases of COVID-19 each month according to self-reported infection status. There is a clear temporal trend such that the proportion of predicted cases increases over time in those who self-reported that they had COVID-19. As noted above, the results from 2019 should be interpreted with caution. Indeed the trends of self-reported infection status are much lower in those months, reflecting the likelihood that the algorithm was picking up a different respiratory infection.

**Table 4.** Predicted cases of COVID-19 each month using Menni *et al*. algorithm ^13^ according to self-reported infection status (note that valid self-report infection status data available for n=6664 which is why the total column is higher than the sum of the other columns).

	Tested positive (0.01%)	Doctor suspected (1.13%)	Participant suspected (12.7%)	Not had (84.6%)	TOTAL
**Oct 2019** n=136	0	1 (0.7%)	40 (29.4%)	95 (69.9%)	**143 (2.10%)**
**Nov 2019** n=64	0	1 (1.6%)	15 (23.4%)	48 (75.0%)	**70 (1.03%)**
**Dec 2019** n=166	0	1 (0.6%)	23 (13.9%)	142 (85.5%)	**176 (2.58%)**
**Jan 2020** n=145	0	0	51 (35.2%)	94 (64.8%)	**151 (2.22%)**
**Feb 2020** n=137	0	3 (2.2%)	56 (40.9%)	78 (56.9%)	**144 (2.11%)**
**March 2020** n=271	0	37 (13.7%)	139 (51.3%)	95 (35.1%)	**285 (4.19%)**
**April 2020** n=153	0	29 (18.9%)	70 (45.6%)	54 (35.3%)	**162 (2.38%)**

Whilst we have presented these results as a mark of the type of analysis one can undertake using these data, we note that these predictions are subject to important assumptions. First, the *baseline risk of having COVID-19* (intercept term in the model) is assumed to be the same in the ALSPAC study population as the Menni study population. This assumption may be invalid as there are fewer reported COVID-19 cases in South West England compared to other regions of the UK ^14^, potentially over-estimating prevalence in ALSPAC. Secondly, the symptoms we termed ‘new persistent cough’, ‘severe fatigue’ and ‘decreased appetite’ are assumed to capture the same information as the symptoms used in the Menni study but may in truth have subtly different meaning, leading to either over or under-estimation of prevalence in the ALSPAC study. Thirdly, the *association of these symptoms with COVID-19* (fixed effects in the model) is assumed to be the same in the ALSPAC study population as the Menni study population. This assumption might be violated if the pattern of symptom presentation varies in different groups of people, leading to over or under-estimation of prevalence in the ALSPAC study.

## Strengths and limitations of the data

The primary strengths of this data are the timelines within which the collection occurred, the retrospectively available and prospectively continuing longitudinal collection forming the context for these new data and the potential for cross-cohort comparisons with a set of measures aligned to other UK studies. We believe the timeframe is important, as the data collected here reflects the feelings of the cohort early on in mitigation during a period of stringent lockdown measures. In addition to this, given that the questionnaire was active for over a month it will be important to take date of completion into account for certain analyses, as responses may differ between early- versus late- completers (e.g. late completers experienced an additional month of lockdown measures and have adapted more to the new circumstances). Our second COVID-19 data collection, funded and planned to start at the end of May will provide invaluable comparisons as (at the time of writing) some lockdown features are starting to be relaxed (certain work sectors being encouraged to return to work etc). Through the Wellcome coordinated group developing a core set of questions we will be able to make valuable comparisons with other cohort groups of different ages, backgrounds and from different countries and cultures. 

We had a good response rate given the pandemic; however, it should be noted that invitations were only sent to those participants for whom we had a valid email address and we therefore have limitations to coverage. Our usual questionnaire strategy is to first send emails to encourage online completion. We then follow up as part of the reminder process and send paper questionnaires through the post. This online only strategy will have particularly affected G0 mothers who historically have tended to use paper questionnaires more than other sub-groups and for whom we are least likely to hold a current email address. This online only strategy will no doubt result in selection bias, which must be taken into account when using this data, as the proportion of original participants in the study taking part is small. Work is ongoing to describe this selection bias and will be published in due course. However, the pandemic has led to a number of participants reaching out and getting in touch to provide these details, and indeed to re-engage with the study having dropped out previously.

In some cases, the data recorded is potentially identifiable. We went through each variable one by one and made decisions about whether to combine categories. We have combined categories where we feel the data is at high risk of potential disclosure.

A further key limitation of this data is the reduced response rate in our male G1 participants (28% of all G1 responders were male), even compared to previous data collection exercises in this group where around a third of G1 responders have been male. 

Finally, the collection was of course subject to the frequency of COVID-19 infection in the population and sample. Very few participants reported a positive test to COVID-19 and the frequency of algorithmically assigned case status suggests a frequency of symptomatic presentation which is consistent with regional estimates ^14^. This is partly due to UK policy not to test widely at the time that data was being collected but also the fact that the majority of our participants remain in the South-West of England which has had the lowest COVID-19 death rate in the country (and potentially the lowest infection rate ^15^. All symptoms were self-reported and the use of the algorithm to predict cases based on these symptoms is subject to a number of limitations as discussed in the results section. We aim to address these limitations by linking to health records (including Public Health England testing results) and direct serological testing of our participants in order to assessthe true symptoms and prevalence of COVID-19 in this population.

## Data availability

### Underlying data

ALSPAC data access is through a system of managed open access. The steps below highlight how to apply for access to the data included in this data note and all other ALSPAC data:

1. Please read the ALSPAC access policy ^16^ which describes the process of accessing the data and samples in detail, and outlines the costs associated with doing so.

2. You may also find it useful to browse our fully searchable research proposals database ^17^, which lists all research projects that have been approved since April 2011.

3. Please submit your research proposal ^18^ for consideration by the ALSPAC Executive Committee. You will receive a response within 10 working days to advise you whether your proposal has been approved.

Please note that a standard COVID-19 dataset will be made available at no charge (see description below); however, costs for required paperwork and any bespoke datasets required additional variables will apply.

## COVID-19 Questionnaire 1 Data File

Data from the first ALSPAC COVID-19 questionnaire is available in two ways.

1. A freely available standard set of data containing *all* participants together with key sociodemographic variables (where available) is available on request (see data availability section). Subject to the relevant paperwork being completed (costs may apply to cover administration) this dataset will be made freely available to any bona fide researcher requesting it. Variable names will follow the format *covid1_xxxx* where *xxxx* is a four-digit number. A full list of variables released is available as extended data ^11^. Frequencies of variable and details of any coding/editing decisions and derived variables are also available in the data dictionary (see extended data ^11^).
2. Formal release files have been created for G0 mothers, G0 fathers and G1 participants in the usual way and now form part of the ALSPAC resource (Due to the small number of G1 partners contributing we will not be formally releasing this data, however, it may be available on request for specific G2 projects). These datasets (or sections therein) can be requested in the usual way. Variable names will replicate those in 1) above but as each variable in ALSPAC is uniquely defined we have added letters to denote the source of the variable. For example, in dataset 1, the age of the participant at completion (in years) is denoted by *covid1_9650*. In the mother’s dataset this will be denoted by *covid1m_9650*, for fathers/partner this will be *covid1p_9650* and for the G1 generation it will be *covid1yp_9650*. Frequencies for all variables for each participant group are available in the data dictionary in the usual way ^19^.

Text data and other potentially disclosive information will not be released until they have been coded appropriately. Table 5 describes the data that is withheld at the time of first release. Text from Section C, question 1 and Section D, other are being thematically coded by qualitative researchers, the remainder will be coded as it is required/requested. Data will be incorporated back into both file sets as they become available.

**Table 5.** Data from questions that will not be released until coded.

Question number	Question text
**Section A**	
1	Please tell us the type of: • Organ transplant • Diabetes • Heart disease or heart problems • Other lung condition • Cancer • Condition affecting the brain and nerves • Psychiatric disorder Please can you tell us why your immune system is weakened?
3	For each medication: • Name of medication • Amount • How often • Reason for taking
5	Which country do you live in? If travelled outside home country: • Country and region/city/resort • Date arrived/left • Purpose of trip
6	What kind of other medical attention did you access? What other medication did you take?
9	Date first told had COVID-19
**Section B**	
1	When did you start self-isolating? Other reason for self-isolating
2	Other reason for changing normal day to day behaviour
**Section C**	
1	What other reason causing worry
**Section D**	
7	Other type of accommodation lived in
Other	Is there anything else you would like to tell us about how the pandemic has affected you?

### Extended data

Open Science Framework: ALSPAC COVID-19 Q1. https://doi.org/10.17605/OSF.IO/ZU8WY ^11^

This project contains the following extended data:

- ALSPAC COVID Q1 FINAL.pdf (The final questionnaire; REDCap PDF)
- ALSPAC_COVID1_varlist.pdf (List of variable names and labels)
- ALSPAC_CovidQ1_data dictionary.pdf (Associated data dictionary including frequencies of all variables that are available)
- ALSPAC_symptom_algorithm_14052020.do (Stata script for obtaining probable COVID-19 cases using the Menni algorithm)

Data are available under the terms of the Creative Commons Attribution 4.0 International license (CC-BY 4.0).

## Consent

Completion of the questionnaire was optional and choosing to complete the questionnaire is considered informed consent for the questionnaire.

Ethical approval for the study was obtained from the ALSPAC Ethics and Law Committee and the Local Research Ethics Committees. Informed consent for the use of data collected via questionnaires and clinics was obtained from participants following the recommendations of the ALSPAC Ethics and Law Committee at the time. Study participants have the right to withdraw their consent for elements of the study or from the study entirely at any time. Full details of the ALSPAC consent procedures are available on the study website ^20^.
