# Supplementary material for: The Avon Longitudinal Study of Parents and Children - A resource for COVID-19 research: Questionnaire data capture April-May 2020
Source: Wellcome Open Res. 2020 Nov 20;5:127. Originally published 2020 Jun 10. [Version 2] doi: 10.12688/wellcomeopenres.16020.2 (PMC7883314; doi:10.12688/wellcomeopenres.16020.2)
Supplement: Peer review history

# Peer review: Reviewer response for version 2

**DOI:** 10.21956/wellcomeopenres.18097.r42302

**Published:** 2021-02-12

**Authors:** Anteo Di Napoli, Martina Ventura

**Affiliations:** 1 National Institute for Health Migration and Poverty (INMP), Rome, Italy

This data note represents a useful instrument to evaluate the relationship between lifestyle, health and biological data, environmental features and COVID-19 infection incidence. Design of Avon Longitudinal Study of Pregnancy and Childhood (ALSPAC) is suitable to follow dynamic changes associated with SARS-COV2 pandemic. In this modified version of the manuscript, the authors improved the first version, responding to all comments from reviewers.

 However, it is important to argue the potential selection bias cited by reviewers, considering the age selection of the ALSPAC study population. This study collects a cohort of pregnant people, their partners and their children from the 90s. As a result, the average age of the cohort is currently about sixty years old. In addition, the use of an emailed questionnaire excluded the older population without a valid email address, which represents the population with high risk of severe form of SARS-COV2 infection. This “age population” selection bias could affect the results of studies that evaluate the association between environment, health, lifestyle, biological data and COVID-19 incidence. The authors should then discuss this issue.

 Lastly, it would be interesting if authors discussed their findings about the Self-Reported Symptoms of SARS-COV2 in the south-west of England comparing them with data from institutional sources, at last for the period from March to April 2020.

Are sufficient details of methods and materials provided to allow replication by others?

Yes

Is the rationale for creating the dataset(s) clearly described?

Yes

Are the datasets clearly presented in a useable and accessible format?

Yes

Are the protocols appropriate and is the work technically sound?

Yes

Reviewer Expertise:

Epidemiology. My expertise is mainly in the field of health inequalities, migrations and nephrology.

We confirm that we have read this submission and believe that we have an appropriate level of expertise to confirm that it is of an acceptable scientific standard.

# Peer review: Reviewer response for version 2

**DOI:** 10.21956/wellcomeopenres.18097.r41525

**Published:** 2020-11-23

**Authors:** Sarah Crozier, Janis Baird

**Affiliations:** 1 MRC Lifecourse Epidemiology Unit, University of Southampton, Southampton, UK

The authors have addressed our previous comments well and we have no further comments to make.

Are sufficient details of methods and materials provided to allow replication by others?

Yes

Is the rationale for creating the dataset(s) clearly described?

Yes

Are the datasets clearly presented in a useable and accessible format?

Yes

Are the protocols appropriate and is the work technically sound?

Yes

Reviewer Expertise:

Sarah Crozier: Associate Professor of Statistical Epidemiology; Janis Baird, Professor of Public Health and Epidemiology, Honorary Consultant in Public Health

We confirm that we have read this submission and believe that we have an appropriate level of expertise to confirm that it is of an acceptable scientific standard.

# Peer review: Reviewer response for version 1

**DOI:** 10.21956/wellcomeopenres.17572.r40286

**Published:** 2020-10-26

**Authors:** Sarah Crozier, Janis Baird

**Affiliations:** 1 MRC Lifecourse Epidemiology Unit, University of Southampton, Southampton, UK

Northstone and colleagues provide a valuable Data Note describing an ALSPAC data-collection wave completed during the COVID-19 pandemic. The ALSPAC team are to be commended for implementing a fast and effective response to a dynamic global situation, allowing prospective data collection at a time of enormous significance. The context of this data resource within a longitudinal study provides great potential for future analyses, and the use of a questionnaire aligned with other longitudinal studies maximises the potential for cross-cohort harmonisation and collaboration.

 The authors have been appropriately cautious to ensure confidentiality in the resource available for release and restriction to non-disclosive variables. Online resources such as the variable list, questionnaire and provision of Stata code will be of great benefit to future researchers. The dataset is large (nearly 7,000 participants). 54% of eligible participants responded to the questionnaire, although the greater proportion of females than males responding could lead to potential biases in some analyses. 

 In the Introduction to the paper, the UK ‘lockdown’ strategy is referenced; for an international audience, it might be helpful to describe what measures were employed by the UK government in March to May 2020 in order to illustrate the restrictions being experienced by cohort members.

 In Table 2 BMI should be summarised using a median and interquartile range since this variable will not be normally distributed in this population.

 Figure 2 requires more comment. The Menni algorithm suggests that 1-2% of participants had probable COVID-19 infection in October, November and December 2020. Whilst it is possible that there were cases of COVID-19 in the UK at this time, it is highly unlikely that the proportion of the population infected was 1-2%. The results, therefore, suggest that other respiratory viruses can mimic the symptoms of COVID-19 and bring into question the applicability of the Menni algorithm to data collected before the pandemic in the UK. The fact that the algorithm was developed on data collected in the spring when COVID-19 levels were high but other respiratory virus levels are likely to have been lower, may be relevant.

 Similarly, further discussion is required regarding the message of Table 4. The percentages suggest that participants who were ill with symptoms resembling COVID-19 in October were less likely to report having ever had suspected COVID-19 than those ill with symptoms resembling COVID-19 in more recent months. The authors could comment further on these trends.  Also, Table 4 would perhaps be more logical, and would certainly follow better from Figure 2, if October 2019 was the first line in the table and April 2020 was the last.

 The term ‘excellent’ (regarding the response rate) on page 7 might be moderated somewhat; the authors should certainly be commended for the success of their data collection within the pandemic situation, but the response rate might not be seen as ‘excellent’ in a broader context. The authors might also note as a limitation that the proportion of total participants responding was not large (i.e. 2,973 G1 responses were received from the original 14,701 children alive at 1 year (20.2%)) leading to potential implications for the representativeness of the responding sample.

**Minor comments:**

  

- In the Abstract the G0 and G1 notation needs to be explained.
- The following sentence in the Abstract is potentially confusing: ‘Using algorithmically defined cases, we estimate that the predicted proportion of COVID-19 cases fell between 1.03% - 4.19% depending on the timing of measurement during the period of reporting.’.  The term ‘fell between’ potentially implies there has been a fall in cases over time which is not what is described.  Also, the period of reporting could be defined fully in this sentence as October 2019 to March 2020.
- Table 1 should be referenced earlier in the paragraph beginning ‘Overall, female participants…’
- Page 4, first paragraph under ‘Key results’: the last sentence here is unnecessary since this has already been stated.
- Page 4, third bullet point under ‘C’: ‘Disorder’ rather than ‘’Disortder’
- Page 8, line 7: Add close bracket.
- Page 8: In the second point describing availability of data, letters (rather than digits) have been added to denote the source of the variable.

Are sufficient details of methods and materials provided to allow replication by others?

Yes

Is the rationale for creating the dataset(s) clearly described?

Yes

Are the datasets clearly presented in a useable and accessible format?

Yes

Are the protocols appropriate and is the work technically sound?

Yes

Reviewer Expertise:

Sarah Crozier: Associate Professor of Statistical Epidemiology; Janis Baird, Professor of Public Health and Epidemiology, Honorary Consultant in Public Health

We confirm that we have read this submission and believe that we have an appropriate level of expertise to confirm that it is of an acceptable scientific standard, however we have significant reservations, as outlined above.

# Response

**Published:** 2020-11-10

**Authors:** Kate Northstone

**Affiliations:** University of Bristol, UK

Thank you for your positive review of our data note. We respond to your queries according to paragraph of your review.

 Paragraph 3: Thankyou for this helpful suggestion. We have provided further detail as suggested on the UK lockdown.

 Paragraph 4: We have replaced mean(sd) with median(IQR) for BMI as suggested

 Paragraphs 5 and 6: Thankyou for these insightful comments. We agree with the suggestion that the algorithm may not be applicable for data collected pre-2020. We considered removing this data all together but believe it is of interest. However, we have added to both the results and discussion sections as suggested by the reviewers. We have also changed the order of Table 4 as recommended.

 Paragraph 7: Fair point! We have amended this and also acknowledge the inevitable selection bias introduced by our online-only strategy.

 Minor comments:  We have taken into account all the minor comments you suggest and amended the text accordingly. Please note we had to make other minor changes to the abstract to be within the word limit.
